# Supplementary material for: Eco-Efficiency of the Fisheries Value Chains in the Gambia and Mali
Source: Foods. 2021 Jul 13;10(7):1620. doi: 10.3390/foods10071620 (PMC8306238; doi:10.3390/foods10071620)
Supplement: Supplementary file 1 [file foods-10-01620-s001.zip › foods-1281105-supplementary.pdf]

Supplementary material for:

Eco-efficiency of the fisheries value chains in Gambia and Mali

Angel Avadí[1,2,*], Ivonne Acosta-Alba[3,4]

[1] CIRAD, UPR Recyclage et risque, F-34398 Montpellier, France

[2] Recyclage et risque, Univ Montpellier, CIRAD, Montpellier, France

[3] CIRAD, UMR Innovation, F-34398, Montpellier, France

[4] Evalivo, Sustainability Assessment of Food Systems, 02100 Saint Quentin, France

* Corresponding author: angel.avadi@cirad.fr, Tel.: +33 4 67 61 59 67

1 Supplementary data on the Gambian fisheries value chain

More details and data are available in the VCA4D project report "Fisheries and aquaculture Value Chain Analysis in The Gambia" (Avadí et al., 2020).

1.1 Detailed value chains mapping

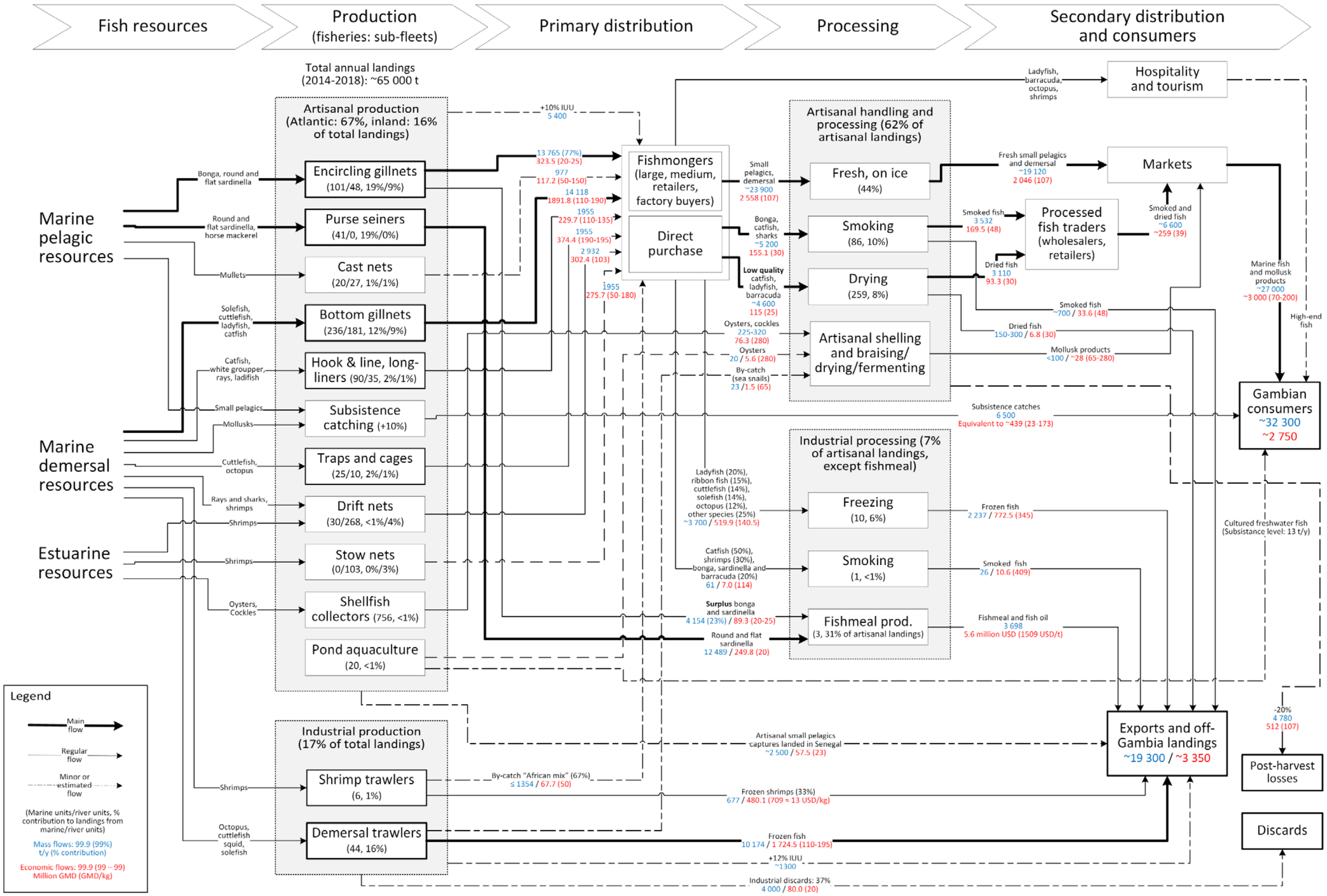

Figure S1. Mapping of the main actors and major material and economic flows in the value chain (detailed)

1.2 LCIs

Table S1. Abridged life cycle inventories of sub-fleets (units of production for fisheries) in The Gambia (average 2014-2018)

		Artisanal units of production								Industrial units of production		
Item	Units	Set/bottom gillnet	Encircling gillnet	Purse seine	Hook and line/ long line	Traps and cages	Drift nets	Cast nets	Stow nets	Shrimp trawlers (SN), 90-230 GRT	Demersal trawlers (CN), 20-90 GRT	Demersal trawlers (CN), 150-400 GRT
Fishery												
Total units	Count	417	149	41	125	35	298	47	103	6	20	29
Atlantic units	Count	236	101	41	90	25	30	20	0	6	20	29
River units	Count	181	48	0	35	10	268	27	103	0	0	0
Target species		Sole, cuttlefish, lobster, ladyfish, catfish	Bonga shad, round and flat sardinella	Round and flat sardinella, horse mackerel	Catfish, white grouper, rays, ladyfish	Cuttlefish, octopus	Rays and sharks (Atlantic), pink shrimps (estuary)	Mullets	Pink shrimps (estuary)	Pink shrimps	Octopus, cuttlefish, squid, sole	Octopus, cuttlefish, squid, sole
Total landings per sub-fleet	t/y	14 118	17 919	12 489	1 955	1 955	2 932	977	1 955	677	1 453	8 721
Fleet motorisation	%	92	93	93	28	92	93	N/A	9	100	100	100
Vessel characteristics												
Dominant build type		Wooden, planked	Wooden, planked	Wooden, planked	Wooden, planked	Wooden, planked	Wooden, planked	N/A	Wooden, planked	Steel	Steel	Steel
Length	m	10	18	22	7	8	18	N/A	5	41	24	41
Width	m	2	5	8	2	2	5	N/A	1	7	N/A	N/A
LSW	kg	3 139	5 323	6 876	2 667	2 718	5 155	N/A	2 316	22 972	7 617	22 665
Engine	HP	22	32	53	15	20	40	N/A	N/A	721	309	1259
FUI	L/t	372	163	104	420	47	441 - 474	N/A	N/A	1553 - 2 213	2 117	2 117
Landings per vessel (incl. by-catch)	t/y	33.9	120.3	304.6	15.6	55.9	9.8	20.8	19.0	282.0	72.7	300.7
By-catch	%	16.6	3.4	-	36.0	-	45.7	-	70.0	66.7	10	10
Fuel	L/y	12 600	19 600	31 800	6 561	2 600	4 667	N/A	N/A	438 025	153 839	636 575

		Artisanal units of production								Industrial units of production		
Item	Units	Set/bottom gillnet	Encircling gillnet	Purse seine	Hook and line/ long line	Traps and cages	Drift nets	Cast nets	Stow nets	Shrimp trawlers (SN), 90-230 GRT	Demersal trawlers (CN), 20-90 GRT	Demersal trawlers (CN), 150-400 GRT
Lubricating oil	L/y	-	-	-	-	-	-	-	-	9.2	9.2	9.2
Ice	t/y	1.1	-	-	1.1	1.1	-	-	-	-	-	-
Bait	t/y	-	-	-	20.3	-	-	-	-	-	-	-
Refrigerant (R22)	kg/y	-	-	-	-	-	-	-	-	0.69	0.69	0.69
Allocation key for main catch	%	88	93	100	67	100	72	100	61	77	93	93
Vessel construction												
Wood	m3	4.0	6.7	8.7	3.4	3.4	6.5	N/A	2.9	0	0	0
Engine	kg	57	97	112	50	57	97	N/A	N/A	1 283	565	2 220
Gear	kg	100	200	500	23	6	400	5	25	4 260	1 098	4 542
Galvanised nails	kg	10.13	17.17	22.18	8.60	8.77	16.63	N/A	7.47	Proxy processes from ecoinvent 3.5 used in the absence of primary data		
Iron rods	kg	284.88	483.02	623.94	241.98	246.67	467.81	N/A	210.20			
Antifouling paint	kg	11.14	18.89	24.40	9.46	9.65	18.30	N/A	8.22			
Tarpaulin	kg	15.24	25.84	33.38	12.95	13.20	25.03	N/A	11.25			
Tar	kg	32.70	55.44	71.62	27.78	28.31	53.70	N/A	24.13			
PET foam	kg	197.51	334.88	432.58	167.77	171.02	324.34	N/A	145.73			

Sources: DoF data, DoF (2016), field visits to landing sites, engine providers, UNEP (2014), field visits to artisanal vessels construction sites. Proxy data for industrial sub-fleets was retrieved from Senegalese shrimp trawlers data (M. Deme; F. Ziegler, pers. comm.) and Mauritanian cephalopods/demersal trawlers (Vázquez-Rowe et al., 2012). Primary data for artisanal driftnets targeting shrimps was complemented with Senegalese data (F. Ziegler, pers. comm.). **Notes:** Landings per fleet and total landings differ slightly from the official figures because by-catch estimations from UNEP (2014) have been added to compute the FUI. By-catch by demersal trawlers was unknown, but assumed at 10% and consisting of small pelagics (which are or should be landed in The Gambia). The demersal trawler fleet was divided in two segments to highlight the size diversity, but not enough data was available to estimate FUI for each segment, thus the whole fleet was modelled as a homogenous type of unit of production. The weighted average of (artisanal) pelagic FUI is 150 L/t, and of demersal is 401 L/t. The weighted average of industrial demersal FUI is 2 055 L/T. The allocation key was computed based on the relative prices of target catch and by-catch (Acosta-Alba and Avadí, 2021). **Abbreviations:** FUI – fuel use intensity, LSW – lightship weight, GRT – gross registered tonnage

Table S2. Abridged life cycle inventories of processing facilities (units of production for processing) in The Gambia (average 2014-2018)

		**Artisanal units of production**		**Industrial units of production**		
Item	Unit	Artisanal fish drying	Artisanal fish smoking	Freezing plants	Smoking plant	FMFO plants
Samples / sites		3/3	3/3	2/1	1/1	3/3
Cold storage capacity	t			580	12	
Fish processed	t/y	21.5	134.8	1 658	61	16 642
Product yield	t/y	7.2	47.2	1 314	26	3 698
Conversion ratio		3.0	2.9	1.3	2.3	4.5
Water	m3/y			33 522	240	29 789 974
Ice	t/y			1 169	6.0	
Salt	kg/y	5 775			200	
Electricity	kWh/y			1 008 846	51 923	254 629
Chemicals (detergents, etc.)	L/y			1 989		22 634
Refrigerating gas (R-22)	kg/y				27.2	
Cardboard	kg/y			6 572	130	
Plastic	kg/y			16 115	20	8 538
Diesel	L/y			25 300	600	
Charcoal	kg/y				6 000	
Coconut husks	kg/y				2 000	
Wood	m3/y		93			
Heavy fuel (R500)	MJ/y					39 941 865
Antioxidants	kg/y					1 664
Wastewater	m3/y			33 522	240	29 789 974
Waste	t/y	1.1	6.7	343	4	

Sources: primary data except for fishmeal plants (FMFO), which was adapted from Fréon et al. (2017)

1.3 Impacts

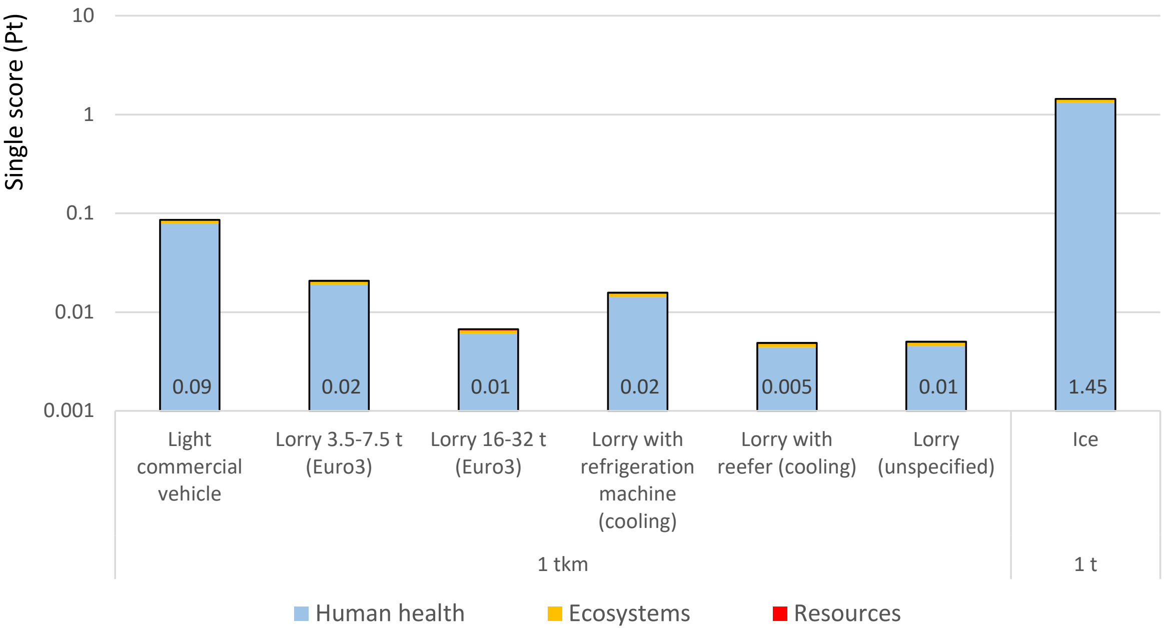

Figure S2. Impacts of transporting 1 tkm of product (log scale), under different vehicle standards, and impacts of producing 1 t of ice in The Gambia

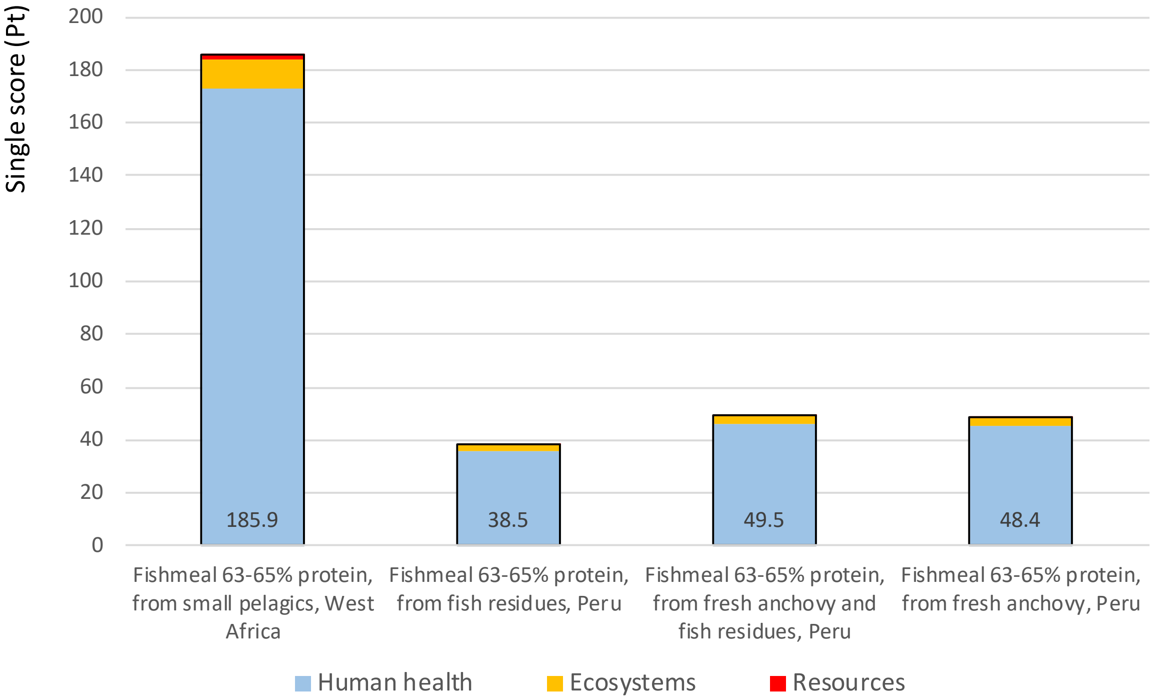

Figure S3. Comparison of West African and global fishmeal production, per t of product and per area of protection

Table S3. LCIA of Gambian fisheries (ReCiPe 2016 Endpoint (H) V1.03 / World (2010) H/A (per Area of Protection) and Cumulative Energy Demand v1.11) including energy (Gross Energy Content) and protein in whole fish

Systems	Species	Total	Human health	Ecosystems	Resources	CED	GEC	Protein	Edible yield
		Pt	Pt	Pt	Pt	MJ	MJ/kg	%	%
Small pelagic fish, capture by artisanal encircling gillnets and landing whole, fresh	Bonga shad, Round sardinella, Flat sardinella	45.7	42.4	2.8	0.5	7 437	7.1	20.7	62.0
Small pelagic fish, capture by artisanal purse seines and landing whole, fresh	Round sardinella, Flat sardinella, Horse mackerel	30.4	28.3	1.8	0.3	4 946	6.8	21.1	60.3
Small pelagic fish, capture by artisanal cast nets and landing whole, fresh	Mullets	0.03	0.02	0.002	0.001	11.7	4.9	20.0	50.0
Demersal fish, capture by artisanal bottom gillnets and landing whole, fresh	Sole, Cuttlefish, Lobster, Ladyfish, Catfish (marine)	114.3	106.2	6.9	1.3	18 578	5.7	20.5	60.0
Demersal fish, capture by artisanal lines and landing whole, fresh	Catfish, White grouper, Rays, Ladyfish	190.0	176.4	11.5	2.1	31 190	6.1	21.2	59.8
Cephalopods, capture by artisanal traps and cages and landing whole, fresh	Cuttlefish, Octopus	13.7	12.7	0.9	0.2	2 407	4.6	17.7	65.5
Demersal fish, capture by artisanal driftnets and landing whole, fresh	Rays, Sharks	190.8	176.7	12.0	2.2	34 871	6.6	21.0	70.0
Shrimp, capture by artisanal stow nets and landing whole, fresh	Pink shrimps	0.9	0.7	0.3	0.01	1 289	5.2	20.4	57.0
Average artisanal-caught pelagic fish	Bonga shad, Round sardinella, Flat sardinella	39.4	36.6	2.4	0.4	6 416	7.1	20.7	62.0
Average artisanal-caught demersal fish	Sole, Cuttlefish, Ladyfish, Catfish (marine), Octopus	121.6	112.9	7.4	1.3	20 353	5.5	19.9	61.6
Shrimp, capture by industrial trawler and landed whole, frozen	Pink shrimps	1 047.6	973.7	62.4	11.4	165 014	5.2	20.4	57.0
Cephalopods and demersal fish, capture by industrial trawler and landed whole, frozen	Cuttlefish, Octopus, Ladyfish, Sole	632.3	587.7	37.6	6.9	99 596	5.4	19.5	64.0

Table S4. LCIA of Gambian fish processing (ReCiPe 2016 Endpoint (H) V1.03 / World (2010) H/A (per Area of Protection) and Cumulative Energy Demand v1.11) including energy (Gross Energy Content) and protein in whole fish

Systems	Species	Total	Human health	Ecosystems	Resources	CED	GEC	Protein
		Pt	Pt	Pt	Pt	MJ	MJ	%
Dried fish (artisanal)	Catfish (marine), Ladyfish	383.1	356.3	22.8	4.0	61 417.9	17.5	61.7
Smoked fish (artisanal)	Bonga shad, Catfish (marine), Sharks	308.4	282.9	22.2	3.3	68 783.1	10.7	33.9
Smoked fish (industrial)	Catfish, Pink shrimps, Bonga shad	274.4	252.0	19.5	2.8	72 927.4	10.7	33.9
Frozen fish (industrial)	Ladyfish, Ribbon fish, Cuttlefish, Sole, Octopus	204.8	190.4	12.4	2.0	39 184.1	5.5	19.4
Fishmeal, 63-65% protein, from small pelagics, West Africa	Round sardinella, Flat sardinella	185.9	172.7	11.3	1.8	32 456.7	17.9	64.0
Shellfish collecting and braising	Oysters, Cockles	64.0	21.0	42.5	0.4	207 560.4	4.7	18.0

2 Supplementary data on the Malian fisheries value chain

More details and data are available in the VCA4D project report “Analyse de la chaîne de la pêche continentale en République du Mali” (Andres et al., 2020).

Table S5. Modelled Malian fishing products volume per year

Products	Total quantity (t)	Fresh equivalent (t)
Fresh fish at fishing zone markets	55 000	55 000
Fresh fish at urban markets	23 000	23 000
Smoked fish at fishing zone markets	6 667	20 000
Smoked fish at urban markets	9 333	28 000
Imported frozen Fish (small pelagics)	64 000	64 000
Imported frozen Fish (fish farming)	16 000	16 000
Export – smoked fish	1 333	4 000
Total		210 000

2.1 LCIs

Table S6. Main characteristics of artisanal fishing units in Mali

Variables by type of fishing unit	Units	Casual (opportunistic) fishers	Agro- (part-time) fishers	Migrant professional (full-time) fishers	Sedentary professional (diversified) fishers
Fishing Units (FU)		178 571	6 387	6 100	31 371
Total annual landings	t	30 000	5 000	29 000	66 000
Landing per FU/year	t	0.168	0.787	4.680	2.100
Fishing season period	months	3	5	12	12
Fishermen per FU		1	3	4.5	3.5
Canoes per UF		-	2	3	2
Self-consumption proportion	%	100	16	11	20
Small sized fish in landings	%		25	25	25
Higher priced fish in landings	%		58	25	38
Proportion of smoked fish per landing	%		17	50	37
Fuel intensity use	l/t	-	96	233	69

Table S7. Abridged life cycle inventories of canoes (per fishing units) in Mali (average 2017-2019)

Variables	Units	Agro- (part-time) fishers	Migrant professional (full-time) fishers	Sedentary professional (diversified) fishers	Description	Ecoinvent background process used (Cut-off, U) market for
Canoe size	m	8	8	8		
Lifetime	yr	7	10	10		
wood	t	1	1	1	African mahogany (Khaya	Occupation, forest, used et Sawnwood, board, softwood, raw, dried (u=20%) {GLO}|

					senegalensis) (density 0.78)	
Imported wood for Canoes	km	1500	1500	1500	80% from Ghana or Ivory Coast	Transport, freight, lorry 16-32 metric ton, euro3 {RoW}| market for transport, freight, lorry 16-32 metric ton, EURO3
Nails	g	500	500	500	Steel	Steel, low-alloyed {GLO}|
Motorized canoes	%	50	50	10		Motor scooter, 50 cubic cm engine {RoW}| motor scooter
Engine power	HP	16	16	16		
Engine lifetime	yr	3	3	3		
Fuel consumption	l/yr	76	941	126	Manufacturing and combustion	Diesel, burned in fishing vessel {GLO}| market for diesel, burned in vessel
Shea butter (manufacture and maintenance of canoes)	kg/yr	35	45	45	Manufacturing Shea is mostly manual, combustion is based on firewood	

Table S8. Abridged life cycle inventories of fishing gear (per fishing units) in Mali (average 2017-2019)

Gears	Units	Casual (opportunist ic) fishers	Agro- (part-time) fishers	Migrant (full-time) fishers	Sedentary (diversified) fishers	Description	Ecoinvent background process used (Cut-off, U)
**Surface gillnets 300m linear**	quantity		3.0	2.6	3.0	Main species caught: *Alestes* and *Brycinus*	
Weight	kg/yr		1.8	1.6	1.8	Nylon (polyamide Polystyrene floats), if recycled material is considered in the end of life	Nylon 6 {GLO}| market for
Lifetime	yr		1.5	2	1.5		
**Bottom gillnets 400m linear**	quantity				5.6	Species caught: *Alestes sp, Brycinus sp, Bagrus sp, Heterobranchus, Clarias sp, Hydrocynus sp, Labeo sp, Lates niloticus, Oreochromis, Sarotherodon, Tilapia*	
Weight	kg/yr				3,2	nylon 80% and polyethylene	Polyethylene et Nylon 6 {GLO}|
Floats						Polystyrene, recycling material not included	
Leads - ballast	quantity				200	(50% terracotta; 50% lead from inside of car batteries recycling), only 10% remains in the water	
Weight	kg/yr				1.0		
Lifetime	yr				1.5		
**Cast nets**	quantity	1	5.0	3	5.0	Main species caught: All species except *Clarias*	
Weight	kg/yr	0.15	0.75	0.45	0.75	Nylon and polyethylene	Polyethylene, low density, {GLO}| and Nylon 6 {GLO}|
Leads - ballast			80	80	80	(50% terracotta; 50% lead from inside of car batteries recycling), only 10% remains in the water	
Weight	kg/yr	0.3	1.32	0.72	1.32		
Lifetime	yr		5	5	5		
**Traps**	quantity	1	4	20	35	Main species caught: *Clarias*	
Weight	kg/yr		0.03	0.06	0.12	Net, nylon spools and kindling	Polyethylene, low density, {GLO}| and Nylon 6 {GLO}| market for
Nylon spools	quantity		1	2	4	210D/45 1/2LB	Nylon 6 {GLO}| market for
Weight	kg/yr		2.5	5	10		
Lifetime	yr		2	2	2		
**Longlines**	quantity		2		4	Main species caught: *Clarias* and *Lates n.*	
Nylon spools	quantity		2		4	210D/45 1/2LB	Nylon 6 {GLO}| market for
Weight	kg/yr		1		2		
Hooks	quantity		4		8	Boxes (1000 units)	Steel, low-alloyed, hot rolled {GLO}| market for
Weight	kg/yr		1.2		2.4		
Lifetime	yr		1		1		

Table S9. Abridged life cycle inventories of transport for imported gear

Variables	units	*Clarias*	*Tilapia*
Type of production		Intensive ponds	Integrated fish farming in ponds
Feed consumption	kg/t of fish	810	1196
Electricity	kWh/t of fish	41	3547
Diesel	l/t of fish	-	74.9
Lime	kg/t of fish	5.2	19

Source: Adapted from Bosma et al. (2011) and Henriksson et al. (2014)

Table S10. Abridged life cycle inventories of transport for imported gear

Products	Type of transport	Origin	Distance (km) One way	Ecoinvent background process used (Cut-off, U)
Wood for canoe	Road	Ivory Coast or Ghana	1 500	Transport, freight, lorry 16-32 metric ton, euro3 {RoW}| market for transport, freight, lorry 16-32 metric ton, EURO3
Gear nets	Ship	Asia (Korea, China, Taiwan)	19 446	Transport, freight, sea, transoceanic ship {GLO}| market for
	Road	Ivory Coast or Ghana	1 500	Transport, freight, lorry 16-32 metric ton, euro3 {RoW}| market for transport, freight, lorry 16-32 metric ton, EURO3

Table S11. Abridged life cycle inventories of collection operations of Malian artisanal fresh fish

Variables	Units	Quantity	Description	Ecoinvent background process used (Cut-off, U)
**Collecting canoe**				
Canoe size	m	15	African mahogany (Khaya senegalensis)	Occupation, forest et Sawnwood, board, softwood, raw, {GLO}|
Lifetime	yr	15		
Load capacity	t	10		
Wood	t	5		
Engine power	HP	35	Manufacture and combustion	Diesel, burned in fishing vessel {GLO}| market for diesel, burned in vessel
Engine Lifetime	yr	5.0		
Fuel consumption	l	15		
Shea butter	kg/yr	45		
**Collector storage**				
Containers	kg	50	Polyurethane	
Weight	g	300		
Lifetime	months	1		
Insulated containers	l	125	Polyurethane from Ghana	Polyurethane Foam PUF
Lifetime	yr	10		
Weight	kg	13		
Recycled refrigerators out of order	units	1	Capacity 300 kg	
**Ice blocks or bags**				

Variables	Units	Quantity	Description	Ecoinvent background process used (Cut-off, U)
Ice in plastic bags from private - individuals	l	1,5	10 bags de 1.5 l per 100kg of fish	
Industrial ice blocks	kg	300	Per 1000 kg de transported fish	
**Market storage**				
Recycled refrigerators out of order (wholesalers)	units	2	Capacity 300 kg	
Industrial ice blocks	kg	25	200 ice blocks	

Table S12. Abridged life cycle inventories of transport operations of Malian fish products

Products	Type of transport	Origin	Distance (km) One way	Ecoinvent background process used (Cut-off, U)
Smoked fish to urban markets	Road	Mopti	635	Transport, freight, lorry 7.5-16 metric ton, euro3 {RoW}| market for
Smoked fish exported	Road	Mopti to Ivory Coast or Ghana	1 500	Transport, freight, lorry 7.5-16 metric ton, euro3 {RoW}| market for
Fresh fish to urban markets	Road	Distance weighted by regional production volume	545	Transport, freight, lorry 7.5-16 metric ton, euro3 {RoW}| market for

Table S13. Abridged life cycle inventories of transport operations of imported frozen fish (small pelagics)

Port of origin	Distance to Bamako (km)	Distance to Dakar (km)	%	Ecoinvent background process used (Cut-off, U)
Mauritania and Senegal	1448		30	Transport, freight, lorry with reefer, freezing {GLO}|
Namibia	1371	5 522	27	
Morocco	1371	2 053	23	

Table S14. Abridged life cycle inventories of transport operations of imported frozen fish (fish farming)

Type of transport	Origin	Destination	Distance (km) One way	Ecoinvent background process used (Cut-off, U)
Ship	China, Vietnam	Senegal	19 446<br>16 800	Transport, freight, sea, transoceanic ship with reefer, freezing {GLO}| market for
Road	Dakar	Bamako	1 200	Transport, freight, lorry with reefer, freezing {GLO}| market for

2.2 Impacts

Table S15. LCIA of Malian fisheries, imported fish (small pelagics and fish farming) (ReCiPe 2016 Endpoint (H) V1.03 / World (2010) H/A (per Area of Protection) and Cumulative Energy Demand v1.11)

Fish production	Total	Human health	Ecosystems	Resources	CED
	Pt	Pt	Pt	Pt	MJ
Casual (opportunistic) Malian fishers	3.0	2.7	0.2	0.1	6.0
Agro- (part-time) Malian fishers	45.8	42.0	3.3	0.6	91.6
Sedentary (diversified) Malian fishers	28.6	26.4	1.9	0.3	57.2
Migrant (full-time) Malian fishers	80.1	74.4	4.8	0.9	160.2

Tilapia extensive fish farming (China)	370.0	316.6	51.9	1.6	740.0
Catfish based in pond (Vietnam)	73.4	56.8	16.2	0.3	146.7
Small pelagic fish. capture by artisanal purse seines (Gambian process)	30.4	28.2	1.8	0.3	60.8
Small pelagic fish. capture by artisanal encircling gillnets (Gambian process)	45.6	42.4	2.8	0.5	91.3
Frozen fish (industrial) - (Gambian process)	217.7	202.1	13.1	2.5	435.4

Table S16. LCIA of Malian artisanal fisheries products and imported fish products (ReCiPe 2016 Endpoint (H) V1.03 / World (2010) H/A (per Area of Protection) and Cumulative Energy Demand v1.11

System	Total	Human health	Ecosystems	Resources	CED
	Pt	Pt	Pt	Pt	MJ
Fresh fish at rural market	15.0	13.8	0.98	0.25	4 731
Fresh fish at urban markets	64.7	59.6	4.12	1.01	18 883
Smoked fish at fishers place	139	129	8.69	1.55	25 882
Smoked fish at urban market	145	134	9.10	1.70	28 270
Smoked fish at export market	152	141	9.62	1.90	31 294
Frozen marine fish imported to Mali	126	116	7.92	1.84	29 590
Frozen *Clarias* imported to Mali (ML) from Vietnam until Bamako	129	108	19.60	1.41	36 795
Frozen *Tilapia* imported to Mali (ML) from China until market Bamako	429	371	55.46	2.69	98 006

References

Acosta-Alba, I., Avadí, A., 2021. Mapping fisheries value chains to facilitate their sustainability assessment: case studies in The Gambia and Mali. Mar. Policy.

Andres, L., Alba, I.A., Nicolay, G., Oswald, M., Zerbo, H., 2020. Analyse de la chaîne de la pêche continentale en République du Mali, Rapport pour l'Union Européenne, DG-DEVCO. Value Chain Analysis for Development Project (VCA4D CTR 2016/375-804), 151 p + annexes. Brussels: European Commission, Directorate-General for International Cooperation and Development.

Avadí, A., Deme, M., Mbaye, A., Ndenn, J., 2020. Fisheries and aquaculture Value Chain Analysis in The Gambia, Report for the European Union, DG-DEVCO. Value Chain Analysis for Development Project (VCA4D CTR 2016/375-804), 139p + annexes. Brussels: European Commission, Directorate-General for International Cooperation and Development.

Bosma, R., Anh, P.T., Potting, J., 2011. Life cycle assessment of intensive striped catfish farming in the Mekong Delta for screening hotspots as input to environmental policy and research agenda. Int. J. Life Cycle Assess. 16, 903–915. https://doi.org/10.1007/s11367-011-0324-4

Department of Fisheries, 2016. The 2016 Frame Survey Report. Banjul: Department of Fisheries.

Fréon, P., Durand, H., Avadí, A., Huaranca, S., Orozco Moreyra, R., 2017. Life cycle assessment of three Peruvian fishmeal plants: Toward a cleaner production. J. Clean. Prod. 145, 50–63. https://doi.org/10.1016/j.jclepro.2017.01.036

Henriksson, P.J.G., Zhang, W., Nahid, S.A.A., Newton, R., Phan, L.T., Dao, H.M., Zhang, Z., Jaithiang, J., Andong, R., Chaimanuskul, K., Vo, N.S., Hua, H. V, Haque, M.M., Das, R., Kruijssen, F., Satapornvanit, K., Nguyen,

P.T., Liu, Q., Liu, L., Wahab, M. a, Murray, F.J., Little, D.C., 2014. Final LCA case study report Primary data and literature sources adopted in the SEAT LCA studies, SEAT Deliverable Ref: D 3.5. Annex report. SEAT: Sustaining Ethical Aquaculture Trade.

UNEP, 2014. Cost-benefit Analysis of the most profitable fishing practices from a societal perspective in The Gambia.

Vázquez-Rowe, I., Moreira, M.T., Feijoo, G., 2012. Environmental assessment of frozen common octopus (Octopus vulgaris) captured by Spanish fishing vessels in the Mauritanian EEZ. Mar. Policy 36, 180–188. https://doi.org/10.1016/j.marpol.2011.05.002
